# Cardio-cerebrovascular adverse outcomes in patients with influenza with and without preexisting cardiovascular disease: Oral antiviral agents impact

**DOI:** 10.1097/MD.0000000000039032

**Published:** 2024-07-19

**Authors:** Kyung-Teak Park, Minjea Choi, Jun Hyung Kim, Ki-Woon Kang

**Affiliations:** a Division of Cardiology, Chung-Ang University Hospital, College of Medicine, Chung-Ang University, Seoul, South Korea; b Division of Cardiology, Chung-Nam University Hospital, Daejeon, South Korea.

**Keywords:** adverse events, cardiology, infectious disease

## Abstract

This study aimed to compare the 2-year cardio-cerebrovascular adverse outcomes of patients with influenza with and without preexisting cardiovascular disease (preCVD) treated with oral antiviral agents in the outpatient clinic. Oral antiviral agents are routinely prescribed to treat influenza infection with a positive rapid-antigen test in the outpatient clinic; however, influenza-associated cardio-cerebrovascular outcomes have not yet been characterized in patients with preCVD treated with oral antiviral agents. Data between 2006 and 2016 were extracted from the National Health Database of South Korea. A total of 865,522 patients with influenza treated with oral antiviral agents were selected in South Korea and classified as preexisting ischemic heart disease (IHD), heart failure (HF), or atrial fibrillation (AF), and 2-year cardio-cerebrovascular outcomes were analyzed using a Cox proportional hazard regression model. Among the participants, 96,433 had preCVD (11.1%; mean age, 46 years) including IHD (86.4%), HF (23.1%), and AF (12.5%). The incidence of new-onset IHD, AF, HF, and death was similar between patients with influenza with and without preCVD. The incidences of IHD and stroke were 0.489 and 0.047 per 100-person year in the preCVD group, respectively. The incidence of cardiovascular mortality was 0.489 per 100-person year in the preCVD group, and the hazard ratio for cardiovascular mortality in the preCVD group was not significantly different from that in patients without preCVD. Based on the national health data, 2-year cardio-cerebrovascular adverse outcomes were not significantly different between patients with and without preCVD treated with oral antiviral agents.

## 1. Introduction

Influenza infection contributes to adverse cardio-cerebrovascular outcomes.^[1,2]^ Although influenza vaccination is highly recommended in high-risk populations with preexisting cardiovascular disease (preCVD) to prevent the occurrence of influenza-associated cardio-cerebrovascular adverse outcomes,^[1–4]^ antiviral influenza therapy could also be critical in patients with preCVD because the influenza viral load might act as a trigger for inflammation and hemostasis imbalance, leading to adverse cardio-cerebrovascular outcomes.^[5]^ In particular, severe influenza infection also has detrimental effects on preCVD, with a risk of cardio-cerebrovascular endothelial dysfunction, plaque formation, and atrial fibrillation (AF) during the postinfection period.^[2,6–8]^ Recently, observational data after antiviral therapy in patients with influenza with preCVD were reported over a 6-month follow-up duration.^[9]^ However, over 6-month of follow-up data on the relationship between treated influenza infection and cardio-cerebrovascular adverse outcomes are limited in preCVD patients treated with oral antiviral agents in the outpatient clinic. Therefore, this study included a nationwide cohort to investigate whether patients with influenza with preCVD treated with oral antiviral agents were significantly associated with 2-year cardio-cerebrovascular adverse outcomes compared with those without preCVD.

## 2. Methods

### 2.1. Study population and data source

This study used data from the National Health Information Database (NHID) from 2006 to 2016. The NHID is a public database based on demographic variables and deaths in the entire South Korean population.^[10]^ The NHID consists of an eligible healthcare utilization database, a 2-year care insurance database, and a healthcare provider database^[10]^ which is based on data collected during the process of claiming healthcare services and includes information on medical records of admission and outpatient usage and prescription records.^[10]^ Data can be accessed from the Health Insurance Data Service (http://nhiss.nhis.or.kr).

In this study, patients with influenza infection above the age of 19 years were selected: diagnostic tool code INFLUENZA TEST CZ394 and diagnosis codes Quan International Statistical Classification of Disease and Related Health Problems, Tenth Revision (ICD-10) J9, J10, and J11 (influenza-related infection) with prescription of an oral antiviral agent in an outpatient clinic (oseltamivir or zanamivir, most commonly prescribed). In South Korea, oral antiviral agents were prescribed based on positive influenza fast diagnostic antigen test results, regardless of the polymerase chain reaction.

### 2.2. Ethics review

The study protocol was reviewed and approved by the Institutional Review Board of the Chung-Ang University Hospital (2110-025-19388) and adhered to the principles of the Declaration of Helsinki.

### 2.3. Definition of preCVD and primary end point events

This study classified preCVD as preexisting ischemic heart disease (IHD), heart failure (HF), and AF. New-onset IHD was defined as follows: a diagnosis code of ICD-10 I21 (acute myocardial infarction), I22 (post-myocardial infarction), I23 (myocardial infarction complication), I24 (other acute IHD), I25 (chronic myocardial infarction), or I20 (angina pectoris) and treatment with coronary bypass graft surgery O1641, primary coronary intervention M6565, or thrombolytic agents (i.e., streptokinase). New-onset HF was defined by a diagnosis code of ICD-10 I50 (HF), and new-onset AF was defined by a diagnosis code of ICD-10 I48 (AF) 30 days after the index date. Finally, 865,522 patients with influenza treated with antiviral agents were selected as study participants.

The index date was defined as the date of the first prescription of oral antiviral agents in the outpatient clinic, and IHD, HF, AF, stroke, and death from cardiovascular events were recorded as cardio-cerebrovascular events and mortality. The dates of cardio-cerebrovascular mortality were obtained using a unique deidentified number for each participant linked to mortality information from the Korean National Statistical Office.

### 2.4. Statistical analysis

A Cox proportional model was used to estimate the hazard ratio and 95% confidence intervals to assess the association between cardiovascular mortality and the risk of preexisting IHD, HF, and AF. Potential confounders were adjusted using multivariate regression models. The level of comorbidities of the participants was assessed using diagnostic codes during the years before the index date using ICD-10.^[11]^ The presence of diseases constituting a previous medical history, including AF, HF, IHD, stroke, chronic kidney disease, diabetes mellitus, and hypertension, was defined on the basis of at least 2 outpatient visits or 1 admission with primary or secondary diagnosis codes. Statistical analyses were performed using SAS (version 9.4; SAS Institute, Cary). Statistical significance was set at *P* < .05

## 3. Results

**Figure 1.**
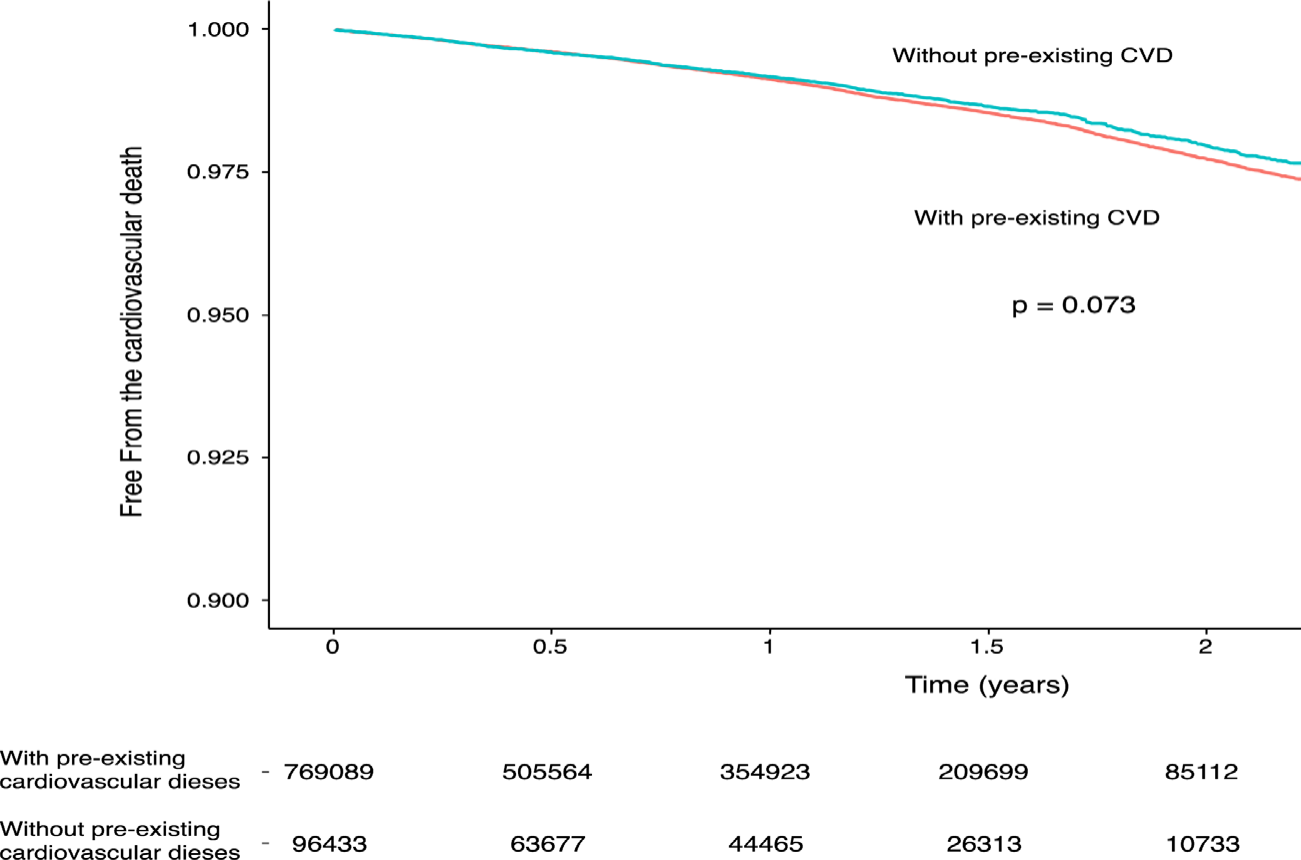
Kaplan–Meier curve compares the free from 2-year cardiovascular death between the 2 groups. CVD = cardiovascular disease.

The proportions of age, sex, and all-cause hospitalization were similar between patients with and without preCVD; however, patients with preCVD had significantly higher proportions of hypertension, diabetes, chronic kidney disease, cancer, and previous stroke (Table 1). The incidences of new-onset AF, new-onset IHD, new-onset HF, all-cause death, and admission to the intensive care unit for influenza were similar between patients with and without preCVD treated with oral antiviral agents (Table 2). The incidences of new-onset IHD and stroke were similar between the 2 groups, regardless of the presence of preCVD, hypertension, diabetes, chronic kidney disease, and cancer (Tables 1 and 2, Supplemental Digital Content, http://links.lww.com/MD/N251, http://links.lww.com/MD/N252). The adverse cardiovascular outcomes in patients with preCVD, including AF, HF, IHD, hypertension, diabetes, chronic kidney disease, and cancer, were not significantly different between patients with and without preCVD in the influenza infection group treated with oral antiviral agents (Table 3; Fig. 1).

**Table 1:** Baseline clinical characteristics of the study participants.

	Without PreCVD (n = 769,089)	With PreCVD (n = 96,433)	*P* value
Age, yr	46.1 ± 15.8	46.1 ± 15.8	.248
Gender, male (%)	472,102 (61.4%)	59,154 (61.3%)	.801
Hospitalization	11,622 (1.5%)	1422 (1.5%)	.388
Ischemic heart disease		83,272 (86.4%)	
Atrial fibrillation		12,032 (12.5%)	
Congestive heart failure		22,287 (23.1%)	
Hypertension	143,097 (18.6%)	73,178 (75.9%)	<.001
Diabetes mellitus	96,021 (12.5%)	46,822 (48.6%)	<.001
Chronic kidney disease	6487 (0.8%)	5286 (5.5%)	<.001
Cancer	179,081 (23.3%)	36,282 (37.6%)	<.001
Stroke	19,673 (2.6%)	16,291 (16.9%)	<.001
Follow-up duration, d	714 ± 550	717 ± 551	.254

preCVD = preexisting cardiovascular disease.

**Table 2:** Incidence of cardio-cerebrovascular events during follow-up.

	Without preCVD (n = 769,089)	With preCVD (n = 96,433)	*P* value
ICU admission	91 (0.0%)	5 (0.0%)	.092
IHD	3851 (0.5%)	471 (0.5%)	.627
AF	11,065 (1.4%)	1357 (1.4%)	.446
HF	146 (0.1%)	15 (0.0%)	.459
Stroke	711 (0.1%)	88 (0.1%)	.953
Cardiovascular Death	7825 (1.0%)	926 (1.0%)	.098

AF = atrial fibrillation, HF = heart failure, ICU = intensive care unit, IHD = ischemic heart disease, preCVD = preexisting cardiovascular disease.

**Table 3:** Incidence of cardiovascular mortality according to risk factors.

	Number of death	Incidence of death (per 100-person year)	Hazard ratio	95% CI	*P* value
Total	8751	0.516			
PreCVD	926	0.489	0.940	0.878–1.006	.073
Gender, male	4063	0.388	0.536	0.514–0.559	<.001
HTN	2202	0.519	1.007	0.959–1.057	.783
DM	1436	0.514	0.994	0.940–1.052	.842
CKD	142	0.607	1.175	0.995–1.052	.057
Cancer	2175	0.515	0.998	0.951–1.048	.935
Preexisting IHD	805	0.493	0.947	0.881–1.018	.143
Preexisting AF	112	0.470	0.905	0.751–1.091	.296
Preexisting HF	211	0.480	0.925	0.807–1.061	.265

AF = atrial fibrillation, CI = confidence interval, CKD = chronic kidney disease, DM = diabetes mellitus, HF = heart failure, HTN = hypertension, IHD = ischemic heart disease, preCVD = preexisting cardiovascular disease.

## 4. Discussion

The results demonstrated that cardio-cerebrovascular adverse outcomes were not significantly different between the treated patients with influenza with and without preCVD in the outpatient clinic during the 2-year observation period.

Influenza infection has been significantly associated with an increased incidence of cardio-cerebrovascular events, including new-onset AF, HF, and IHD.^[3]^ Recently, the proportion of cardio-cerebrovascular adverse outcomes in patients with preexisting comorbidities was significantly higher in hospitalized patients with influenza infection than in those without comorbidities in a short-term observation.^[12]^ Although each seasonal influenza strain has different epidemiological and pathological characteristics, underlying medical comorbidities may be significant risk factors for cardio-cerebrovascular adverse outcomes in the post-influenza period.^[1]^ Influenza-associated immunological processes may also lead to adverse cardio-cerebrovascular outcomes, including endothelial dysfunction, atherosclerotic plaque destabilization, and IHD progression in the postinfection period.

However, at the top of vaccination, the 2-year follow-up observation data of cardio-cerebrovascular events and mortality in influenza outpatients treated with oral antiviral agents have been limited in patients with preCVD according to large-scale national data. In our results, 2-year cardio-cerebrovascular events and mortality were not significantly different between patients with influenza with preCVD treated with antiviral therapy and those without preCVD, and the incidence of intensive care in patients with influenza with preCVD was similar to that in those without preCVD (Tables 2 and 3). In this population-based analysis of patients with influenza treated with oral antiviral agents, preCVD might not have a decremental effect on the occurrence of upcoming cardio-cerebrovascular events and mortality in the 2-year observation, and preCVD might not be a significant risk factor in patients with influenza treated with antiviral agents for the 2-year occurrence of cardio-cerebrovascular outcomes. Therefore, antiviral therapy could be critical in high-risk populations with preCVD to prevent cardio-cerebrovascular complications in the long term.

This study has several limitations. First, the results cannot be generalized to all cases of influenza because recurrent influenza episodes and other antiviral agents caused by annual seasonal epidemics were not interpreted in the present study. Second, none of the patients diagnosed with influenza infection using genetic diagnostic tests may have been included because the study population was extracted based only on influenza diagnostic codes. Third, the NHID also depends on the ICD-10 codes; thus, preCVD may be misdiagnosed, including AF, IHD, and HF. Therefore, verifying the clinical diagnosis or the type and duration of AF, IHD, or HF was impossible using propensity score matching. Fourth, even if influenza vaccination is mandatory for high-risk patients in South Korea, unvaccinated patients with influenza may exist. This study could not investigate the influence of vaccination or reinfection on cardio-cerebrovascular events and death because this information could not be extracted from the national population database.

## 5. Conclusion

Based on the national health information data, 2-year cardio-cerebrovascular events and mortality in patients with influenza with preCVD treated with oral antiviral agents were not significantly different from those without preCVD.

## Acknowledgments

We would like to thank Dr Jinseop Kim (Zarathu Co., Ltd., Seoul, Republic of Korea) for consultaton and MetaBio Co. Ltd., Yongin, Republic of Korea, for advice and financial support.

## Author contributions

**Data curation:** Kyung-Teak Park, Jun Hyung Kim, Ki-Woon Kang.

**Formal analysis:** Kyung Teak Park, Jun Hyung Kim, Ki-Woon Kang.

**Writing – original draft:** Kyung-Teak Park, Minjea Choi, Jun Hyung Kim, Ki-Woon Kang.

**Project administration:** Minjea Choi.

**Resources:** Minjea Choi.

**Software:** Minjea Choi.

**Validation:** Minjea Choi.

**Visualization:** Minjea Choi.

**Conceptualization:** Ki-Woon Kang.

**Funding acquisition:** Ki-Woon Kang.

**Methodology:** Ki-Woon Kang.

**Supervision:** Ki-Woon Kang.

**Writing – review & editing:** Ki-Woon Kang.

## Supplementary Material




