# Supplementary material for: Cardio-cerebrovascular adverse outcomes in patients with influenza with and without preexisting cardiovascular disease: Oral antiviral agents impact
Source: Medicine (Baltimore). 2024 Jul 19;103(29):e39032. doi: 10.1097/MD.0000000000039032 (PMC11398820; doi:10.1097/MD.0000000000039032)
Supplement: Supplementary file 1 [file medi-103-e39032-s001.docx]

**Supplemental Table 1. Incidence of ischemic heart disease in the study participants**

|  | **Number of IHD** | **Incidence of IHD**  **(Per 100-person year)** | **Hazard ratio** | **95% C.I.** | ***P*** |
| --- | --- | --- | --- | --- | --- |
| Total | 4322 | 0.516 |  |  |  |
| PreCVD | 926 | 0.489 | 0.94 | 0.878-1.006 | 0.073 |
| Sex (Male) | 4063 | 0.388 | 0.536 | 0.514-0.559 | <0.001 |
| HTN | 1086 | 0.257 | 1.005 | 0.938-1.077 | 0.881 |
| DM | 702 | 0.252 | 0.982 | 0.906-1.064 | 0.656 |
| CKD | 61 | 0.262 | 1.022 | 0.794-1.316 | 0.867 |
| Cancer | 1055 | 0.251 | 0.974 | 0.909-1.044 | 0.459 |
| Pre-existing IHD | 805 | 0.493 | 0.947 | 0.881-1.018 | 0.143 |
| Pre-existing AF | 112 | 0.470 | 0.905 | 0.751-1.091 | 0.296 |
| Pre-existing HF | 211 | 0.480 | 0.925 | 0.807-1.061 | 0.265 |

C.I., confidence interval; preCVD, pre-existing cardiovascular disease; HTN, hypertension; DM, diabetes mellitus; CKD, chronic kidney disease; IHD, ischemic heart disease; AF, atrial fibrillation; HF, heart failure.
