# Supplementary material for: Cardio-cerebrovascular adverse outcomes in patients with influenza with and without preexisting cardiovascular disease: Oral antiviral agents impact
Source: Medicine (Baltimore). 2024 Jul 19;103(29):e39032. doi: 10.1097/MD.0000000000039032 (PMC11398820; doi:10.1097/MD.0000000000039032)
Supplement: Supplementary file 2 [file medi-103-e39032-s002.docx]

**Supplemental Table 2. Incidence of stroke in the study participants**

|  | **Number of Stroke** | **Incidence of Stroke**  **(Per 100-person year)** | **Hazard ratio** | **95% C.I.** | ***P*** |
| --- | --- | --- | --- | --- | --- |
| Total | 799 | 0.047 |  |  |  |
| PreCVD | 88 | 0.047 | 0.983 | 0.788-1.227 | 0.881 |
| Sex  (Male) | 420 | 0.040 | 0.684 | 0.595-0.786 | <0.001 |
| HTN | 201 | 0.047 | 1.007 | 0.858-1.181 | 0.936 |
| DM | 150 | 0.054 | 1.170 | 0.980-1.398 | 0.082 |
| CKD | 14 | 0.060 | 1.273 | 0.751-2.160 | 0.370 |
| Cancer | 206 | 0.049 | 1.048 | 0.895-1.228 | 0.370 |
| Pre-existing IHD | 83 | 0.051 | 1.085 | 0.864-1.362 | 0.483 |
| Pre-existing AF | 7 | 0.029 | 0.618 | 0.294-1.301 | 0.206 |
| Pre-existing HF | 17 | 0.039 | 0.815 | 0.504-1.318 | 0.405 |

C.I., confidence interval; preCVD, pre-existing cardiovascular disease; HTN, hypertension; DM, diabetes mellitus; CKD, chronic kidney disease; IHD, ischemic heart disease; AF, atrial fibrillation; HF, heart failure; ICU, intensive care unit
